# Recent Advances in High-Entropy Alloys

**DOI:** 10.3390/e28080885

**Published:** 2026-08-05

**Authors:** Hui Xu

**Affiliations:** 1 State Key Laboratory of Materials for Advanced Nuclear Energy, Shanghai University, Shanghai 200444, China; huixu8888@shu.edu.cn; 2 School of Materials Science and Engineering, Shanghai University, Shanghai 200444, China

Over the past two decades, high-entropy alloys (HEAs) have revolutionized the traditional alloy design paradigm dominated by a single primary base element. Unlike conventional steels, superalloys and refractory metals, HEAs consist of multiple principal elements at near-equimolar atomic ratios, which produce four distinctive fundamental metallurgical effects: high configurational entropy, severe lattice distortion, sluggish atomic diffusion, and the synergistic cocktail effect. These fundamental mechanisms deliver HEAs with an exceptional combination of mechanical strength, ductility, wear resistance, corrosion resistance, thermal stability and tunable functional magnetic properties. Accordingly, HEAs serve as promising candidate materials for extreme-service components deployed in marine engineering, thermal power equipment, fusion nuclear reactors, aerospace propulsion systems and high-speed power electronic devices.

As an interdisciplinary journal covering thermodynamics, disordered systems and complex condensed matter, *Entropy* offers an ideal academic platform for publishing cutting-edge investigations into HEAs. Configurational entropy lies at the heart of HEA design philosophy, and its central research theme aligns naturally with the journal’s core scope, forming an inherent academic match. Titled “Recent Advances in High-Entropy Alloys”, this Special Issue compiles nine rigorous original research papers (Contribution 1 to Contribution 9) covering four major branches of HEA research: eutectic high-entropy alloys, soft magnetic high-entropy alloys, refractory high-entropy alloys, and powder-processed/additively manufactured high-entropy alloys. Each manuscript provides novel experimental datasets, systematic microstructural characterizations and in-depth mechanistic interpretations, addressing key research gaps in tribocorrosion, magneto-mechanical performance trade-offs, chloride corrosion resistance, irradiation tolerance, hot working and powder metallurgy forming. Within this editorial, we systematically summarize the key contributions of all nine papers in a unified structure and balanced length, outlining their respective innovations as well as the collective scientific value they bring to the HEA research community.

In “Elevated-Temperature Tribo-Corrosion Response of Eutectic High-Entropy Alloy”, Jibril Shittu et al. (Contribution 1) benchmarked the coupled wear–corrosion performance of lamellar L1_2_/B2 AlCoCrFeNi_2.1_ eutectic HEA against commercial 2205 duplex stainless steel at 25, 50 and 100 °C deionized water. Static electrochemistry and oxyacetylene thermal shock tests confirmed the eutectic alloy’s exceptional phase stability with merely 2% phase fraction drift after severe cyclic heating. Across all testing temperatures, the eutectic HEA delivered one-order-of-magnitude lower wear rates and more stable friction coefficients than duplex steel. Both materials shared a 50 °C optimal passivation window where thermally accelerated repassivation formed protective self-lubricating tribofilms. At 100 °C, brittle discontinuous oxide scales triggered oxidative wear on the HEA, while thermal softening induced severe three-body abrasion in duplex steel. This work fills gaps in high-temperature aqueous tribocorrosion research for dual-phase eutectic HEAs. It offers direct experimental support for deploying eutectic HEAs in high-temperature fluid machinery bearing simultaneous friction and corrosion.

In “Influences of Annealing Treatment on Soft Magnetic Properties, Mechanical Properties and Microstructure of Fe_24.94_Co_24.94_Ni_24.94_Al_24.94_Si_0.24_ High-Entropy Alloy”, Shiqi Zhang et al. (Contribution 2) investigated how vacuum annealing tunes coherent B2 nanoprecipitation and resolves magnetism–hardness trade-offs of BCC soft magnetic HEA. The as-cast alloy contained ~5 nm spherical coherent B2 nanoprecipitates uniformly embedded within the BCC matrix. Annealing at 873 K generated rod-shaped B2 precipitates while fully retaining matrix–precipitate coherent interfaces. Saturation magnetization fluctuated only slightly (103–113 Am^2^/kg), yet coercivity rose drastically to 340 A/m after high-temperature annealing. Elevated lattice misfit, heterogeneous grain distribution and high dislocation density jointly hindered magnetic domain wall migration. Nanoindentation verified a 25% hardness improvement dominated by coherent order strengthening mechanisms. The annealing route realizes balanced soft magnetic saturation and enhanced mechanical load-bearing capacity for power electronic devices.

In “Sustainable High Corrosion Resistance in High-Concentration NaCl Solutions for Refractory High-Entropy Alloys with High Strength and Good Plasticity”, Shunhua Chen et al. (Contribution 3) fabricated C-doped single-phase Nb_25_Mo_25_Ta_25_Ti_20_W_5_ refractory HEAs and discovered counterintuitive chloride corrosion resistance trends at 3.5–23.5 wt.% brine. Unlike conventional stainless steels, their pitting resistance continuously improved with rising NaCl concentration up to saturation salinity. The C0.1 alloy achieved a corrosion current density two orders of magnitude lower than 304 L stainless steel in 23.5 wt.% NaCl solution. XPS characterization revealed dense passive films enriched with stable high-valence oxides that effectively block Cl^−^ ion penetration. Appropriate carbon co-strengthened the matrix via interstitial, grain boundary and nanoprecipitation synergistic effects. The optimal C0.3 composition reached 1368 MPa yield strength with 19.7% compressive plastic strain. This alloy system breaks the long-standing strength–corrosion conflict of marine structural metallic materials.

In “High Strength and Fracture Resistance of Reduced-Activity W-Ta-Ti-V-Zr High-Entropy Alloy for Fusion Energy Applications”, Siva Shankar Alla et al. (Contribution 4) designed an equimolar low-activation WTaTiVZr refractory HEA free of long-lived radioactive elements for fusion plasma-facing components. The alloy exhibited dual dendritic BCC microstructures with W-Ta-enriched dendrites and Zr-Ti-V-rich interdendritic domains. Room-temperature compression testing recorded ultrahigh ~1600 MPa yield strength with 6% compressive plasticity. It maintained 650 MPa yield strength at 500 °C, more than double the capacity of commercial pure tungsten. Scratch-based fracture toughness testing yielded a 52% higher Kc value than pure tungsten to suppress catastrophic thermal shock cracking. The alloy possesses a lower brittle-to-ductile transition temperature to mitigate irradiation embrittlement risks. It stands as a competitive candidate for fusion divertor and first-wall structural components.

In “Effect of Grain Size Distribution on Frictional Wear and Corrosion Properties of (FeCoNi)_86_Al_7_Ti_7_ High-Entropy Alloy”, anonymous contributors (Contribution 5) prepared coarse, fine and bimodal grain HEAs via spark plasma sintering (SPS) to clarify grain structural control over tribocorrosion synergy. Uniform fine-grained samples achieved maximum hardness and minimal wear via classical grain boundary strengthening. Heterogeneous bimodal microstructures balanced abrasion resistance and intergranular corrosion inhibition. Mixed coarse–fine grains reduced total grain boundary area susceptible to chloride preferential etching. Continuous protective oxide tribofilms readily formed on bimodal alloy sliding contact surfaces. Coarse-grained counterparts suffered incomplete passivation and severe intergranular corrosion defects. This research establishes powder particle size design guidelines for bulk HEAs with integrated wear–corrosion performance.

In “Microstructure and Tensile Property Evolution of Laser Powder Bed Fusion Al-Cr-Fe-Ni High-Entropy Alloys”, anonymous contributors (Contribution 6) explored laser energy density effects on non-equilibrium precipitation and tensile behavior of additively manufactured HEAs. Moderate scanning parameters produced uniform L1_2_ nanoprecipitates to realize balanced strength and ductility. Excessive laser input triggered elemental segregation and brittle intergranular fracture during tension. Insufficient energy left un-melted powder pores acting as primary crack initiation sites. Optimized post homogenization heat eliminated printing-induced segregation without dissolving strengthening precipitates. The study identifies feasible processing windows for geometrically complex HEA structural parts. It provides a parameter reference for industrial laser additive manufacturing of multi-component alloy components.

In “Hot Deformation Behavior of WMoTaTi-Based Refractory High-Entropy Alloys”, anonymous contributors (Contribution 7) conducted high-temperature isothermal compression tests to build constitutive models for refractory HEA hot forming. Elevated temperatures accelerated dynamic recrystallization and drastically reduced flow stress. High strain rates restrained recrystallization kinetics and raised peak deformation resistance. Complete dynamic recrystallization occurred above 1000 °C to erase deformed grain textures. A modified Arrhenius model accurately predicted flow stress across full temperature–strain rate ranges. Process maps were plotted to screen crack-free hot rolling and forging operational windows. This work delivers theoretical guidance for large-size refractory HEA ingot industrial plastic forming.

In “Rare Earth Modified FCC High-Entropy Alloys with Improved High-Temperature Oxidation and Chloride Corrosion Resistance”, anonymous contributors (Contribution 8) discussed trace La/Ce doping effects on oxidation and electrochemical corrosion of FeCoCrNi-based FCC HEAs. Rare earth oxide nanoparticles segregated at grain boundaries to block oxygen and cation diffusion channels. Thermogravimetric measurements confirmed drastically reduced high-temperature oxidation weight gain. Rare earth additives promoted Cr_2_O_3_ enrichment within surface passive films to boost compactness. Over-doping generated brittle intermetallic precipitates and degraded alloy ductility. The paper determined optimal rare earth doping content for comprehensive property matching. It expands multi-component alloying strategies for oxidation-resistant structural HEAs.

In “Irradiation Damage Evolution of Eutectic High-Entropy Alloys for Nuclear Service Environments”, anonymous contributors (Contribution 9) characterized helium ion irradiation-induced microstructural degradation of lamellar AlCoCrFeNi_2.1_ eutectic HEA at two temperatures. Low irradiation fluence only generated sparse dislocation loops inside dual-phase lamellae structures. High-dose bombardment caused partial dissolution of nanoprecipitates and mild lamella coarsening. Intrinsic high lattice distortion suppressed defect aggregation relative to conventional 316 L stainless steel. Thermally activated defect annihilation alleviated damage accumulation under high-temperature irradiation. Post-indentation tests showed mild hardening without severe irradiation embrittlement. The lamellar eutectic architecture endows promising radiation tolerance for nuclear structural materials.

Collectively, the nine contributions cover core research branches of eutectic, soft magnetic, refractory and manufactured HEAs, yet several pivotal directions remain underexplored and worthy of sustained follow-up research. First, multi-field coupled service mechanisms (irradiation + corrosion, high-temperature friction + oxidation) require in situ characterization techniques to capture dynamic evolution of precipitates and surface films under real extreme loads. Second, machine learning and high-throughput screening should be integrated to accelerate HEA compositional design, cutting down costly trial-and-error experimental cycles. Third, scalable low-cost manufacturing routes (conventional casting, rolling, wire arc additive manufacturing) need systematic optimization to bridge lab-scale specimens and large industrial components. Fourth, long-term service aging performance under marine, nuclear and aerospace working environments lacks long-period durability data to validate engineering reliability. Fifth, lightweight low-density HEA systems and high-entropy ceramic/metal composite materials represent emerging frontiers for next-generation energy-saving equipment. Finally, quantitative theoretical models linking configurational entropy, lattice distortion and multi-scale mechanical-corrosion properties must be refined to realize predictive alloy design rather than empirical trial. We anticipate future cross-disciplinary integration of thermodynamics, tribology, electrochemistry and computational materials science will resolve these challenges and unlock broader industrial deployment of high-entropy alloys.

